# Annual Address before the Philadelphia Medical Society

**Published:** 1861-01

**Authors:** R. La Roche


Annual Address before the Philadelphia Medical Society. By Dr. R. La Roche.—In place of a brief notice of this classical and instructive address, we would gladly enlarge on its chief features, and draw not a little from its pages, were we allowed to do so by the exigency of the occasion, which precludes further talk in the present number of our journal. The theme chosen by the author is the journey of Horace from Rome to Brundusium, narrated by the poet in the fifth Satire of the first Book. Seldom, if ever, was there so illustrious a company of travelers as in this instance, when Horace was associated with Virgil, L. Varius, and Plotius Tucca, the two latter of whom enjoyed a reputation as poets which "nearly equaled at the time, at least, that of their companions." But the journey was not undertaken by these favorites of the Muses alone and for their gratification in quest of the picturesque and sublime. They were adopted for their agreeable qualities by Mæcenas, Coccius, and Fonteius Capilo, who were sent to Brundusium as peace commissioners, to heal the differences between Augustus and Antony.

Dr. La Roche did not deem it advisable to make the Satire in which Horace gives an itinerary of his travel as only Horace could do, a text for commentary on the freshness and originality of the great Roman poet, and of the subtle volatile essence with which his poetry is pervaded, nor the illustration of his life and character by his own works. Our author has left it to others to show Horace in the light of a truly refined Epicurean philosopher and an advocate of temperance in its largest and most comprehensive sense. His leading canon of taste—the *modus in rebus*, is also applicable to ethics and to the pursuit of all the enjoyments of life; it shows itself, as a late critic happily says, in the "mixture of critical severity and charitable toleration which went to make up Horace's character as a man." The address is chiefly taken up with an examination of the route of travel of the eminent Roman party from Rome to Brundusium in its medico-topographical and associated archæological relations. The road traverses the southern part of the Campagna, and nearly the whole length of the Pontine Marshes, and naturally invites to a disquisition on the successive changes which these regions have undergone in cultivation, population, and health, during the ante-Roman, the ancient Roman, and the modern Roman periods. The taint of impure air has always manifested itself in different districts and spots in these regions; sometimes extensive and virulent when the cultivation of the soil and with it concomitant draining was neglected; and at other times rendered almost harmless by its extreme circumscription under better hygienic conditions in the face of the country, and by the industry of the people inhabiting it. The great central locality for periodical fevers and their common adjuncts was the Pontine Marshes, which have baffled consuls, emperors, and popes in all their efforts and engineering skill to drain them to the desired point of desiccation and to fit them for the hands of the husbandman and the shepherd. Partial success has attended at different times the labors of these rulers, and if we were to designate one as more eminently successful in this respect it would be Pope Pius VI. The entire subject is carefully scrutinized by Dr. La Roche in his customary careful spirit of research and painstaking criticism. In one of the *apropos* in the address, all of which we receive in good part, the author gives a sketch of the topography of ancient Rome, and the spots within the city walls which were most liable, from their low and primarily marshy condition, to produce disease. By the large majority of his readers the details will be found to be as new as they are instructive. The address closes with an inquiry into the extent to which mineral waters were known to and used by the Romans.

